# Alternative interpretations for decreasing voltage with increasing charge in ferroelectric capacitors

**DOI:** 10.1038/srep20825

**Published:** 2016-02-11

**Authors:** Seul Ji Song, Yu Jin Kim, Min Hyuk Park, Young Hwan Lee, Han Joon Kim, Taehwan Moon, Keum Do Kim, Jung-Hae Choi, Zhihui Chen, Anquan Jiang, Cheol Seong Hwang

**Affiliations:** 1 Department of Material Science & Engineering and Inter university Semiconductor Research Center, Seoul National University, Seoul 151-744, Republic of Korea; 2 Electronic Materials Research Center, Korea Institute of Science and Technology, Seoul 136-791, Republic of Korea; 3 State Key Laboratory of ASIC & System, School of Microelectronics, Fudan University, Shanghai 200433, China

## Abstract

Recent claim on the direct observation of a negative capacitance (NC) effect from a single layer epitaxial Pb(Zr_0.2_,Ti_0.8_)O_3_ (PZT) thin film was carefully reexamined, and alternative interpretations that can explain the experimental results without invoking the NC effect are provided. Any actual ferroelectric capacitor has an interfacial layer, and experiment always measures the sum of voltages across the interface layer and the ferroelectric layer. The main observation of decreasing ferroelectric capacitor voltage (V_F_) for increasing ferroelectric capacitor charge (Q_F_), claimed to be the direct evidence for the NC effect, could be alternatively interpreted by either the sudden increase in the *positive* capacitance of a ferroelectric capacitor or decrease in the voltage across the interfacial layer due to resistance degradation. The experimental time-transient V_F_ and Q_F_ could be precisely simulated by these alternative models that fundamentally assumes the reverse domain nucleation and growth. Supplementary experiments using an epitaxial BaTiO_3_ film supported this claim. This, however, does not necessarily mean that the realization of the NC effect within the ferroelectric layer is impractical under appropriate conditions. Rather, the circuit suggested by Khan *et al*. may not be useful to observe the NC effect directly.

Although ferroelectric switching is one of the most significantly researched topics in solid state physics, it is still an intriguing research area for modern electronic devices. One of the most arguable comments recently reported is the involvement of a negative capacitance (NC) effect in ferroelectrics in a dielectric (DE) – ferroelectric (FE) stacked system^1^,^2^. The fundamental assertion of the NC effect from the FE materials is ascribed to negative coefficient (α) of P^2^ term in the Landau-Ginzburg-Devonshire expansion of free energy equation (U = αP^2^ + βP^4^ + γP^6^), where P is the polarization, represented by a negative slope in its polarization – voltage (P-V) curve. Figure 1 shows a schematic P-V diagram showing two paths that a FE film may take when it is switched from negative polarization state (−P_r_ state) to positive polarization state (+P_s_ state). Along the path 1, the FE film does not form a domain structure but uniformly changes its polarization from −P_r_ to +P_s_ state through origin of the P-V graph, so it does not involve any domain boundary related energy. Near P and V = 0, the FE film is in the NC state. However, it should go through the maximum U state near P = 0, indicated by the U-P graph in the figure, where the energy cost is usually higher than the energy related with domain boundaries. Therefore, realization of the NC effect (negative slope portion of the P-V curve) from the single FE layer has been hampered due to the involvement of the domain formation, and a hysteretic P-V curve has generally been observed (path 2 in Fig. 1). In both cases (path 1 and path 2), positive charge moves into the FE capacitor, but for path 1 there is a region where the voltage decreases. In fact, observing the NC effect from a single FE layer is considered to be very challenging because it means that the capacitor should go through the maximum energy state during its polarization switching. Therefore, the experimental proof of the emergence of the NC effect from a FE layer has been accomplished by measuring the increased capacitance of a FE layer with a DE layer stacked on top^3^,^4^,^5^. In this case, the overall capacitance could still be remained in a positive region, meaning that the experimental proof may not involve any conceptual difficulty. Nevertheless, extremely high capacitance density (C_total_) which is most likely to occur when the absolute values of the NC and PC match each other (C_total_^−1^ = C_PC_^−1^ − C_NC_^−1^, meaning that the total capacitance is ∞ when the absolute magnitude of the NC equals to that of the PC) has not been reported yet. While these are intriguing experimental accomplishments, a direct proof of the emergence of the NC effect from a single layer FE film, i.e. the observation of the decreasing capacitor voltage with increasing charges, is still remained as an impending task to directly prove the NC effect from the FE layer.

Recently, Khan *et al*. reported an eye-catching work about a direct observation of the negative capacitance effect from a 60nm-thick epitaxial single layer Pb(Zr_0.2_,Ti_0.8_)O_3_ (PZT) thin film on SrRuO_3_ electrode/SrTiO_3_ substrate^6^. In that work, they connected the FE capacitor to the voltage source via an external series resistor (R, 50 kΩ, see Fig. 2b of ref. 6), and monitored the variations of the FE capacitor voltage (V_F_) and charges (Q_F_) simultaneously. This is a plausible approach to observe the charge and voltage variations on the FE capacitor in time domain because the adopted R significantly limits the polarization compensating current flow. Their main NC claim stems from both the decrease in V_F_ and the concurrent increase in Q_F_ estimated from the current flow through R (i_R_), upon a voltage pulse application which is high and long enough to induce the FE switching. They also presented theoretical simulations supporting the claim based on Landau-Khalatnikov (L-K) formalism. Conventional FE switching is mediated by the reverse domain nucleation and growth requiring rapid supply of compensating charges from a voltage source, which can be represented by path 2 of Fig. 1. This process does not involve any NC effect. However, the adoption of large R in the circuit of ref. 6 could retard the compensating charge supply, making the FE switching may follow the path 1 of Fig. 1. Nonetheless, the high energy cost involved in this process, i. e. the FE capacitor should go through the maximum U point during the FE switching, may make this process unlikely to occur. Another difficulty encountered in the work of Khan *et al*. was that the calculated coercive voltage (V_c_, ~10 V, in Fig. 4 of ref. 6) based on L-K model was much higher than the experimental result (~3.1V). According to Fig. 1, the V_c_ for path 2 (reverse domain nucleation and growth) is always smaller than path 1 (NC effect), so the experimental result of Khan *et al*. might be better explained by path 2. Therefore, the authors provide alternative models that can well explain the V_F_↓Q_F_↑ behavior without invoking the NC effect as follows.

## Results and Discussion

The important concept for these alternatives is that any actual FE capacitor is almost always accompanied with in-series resistance component (R_i_), which could be interfacial dead-layer or any other non-FE layer. In ref. 6, Khan *et al*., took this into account by introducing an internal resistor (ρ), which is identical to the R_i_ in their circuit model. ρ in ref. 6 was taken to be a constant during the FE switching, but R_i_ is this work is voltage- and electrical stress-dependent. In the interpretation of V_F_↓Q_F_↑ behavior of the circuit in ref. 6, the voltage on the actual FE layer (V_int_) was assumed to decrease during FE switching according to the L-K formalism, which resulted in the increase of the voltage and the current across R. However, the authors took an alternative view on the roles of each component in the circuit as follows.

**Figure 2:**
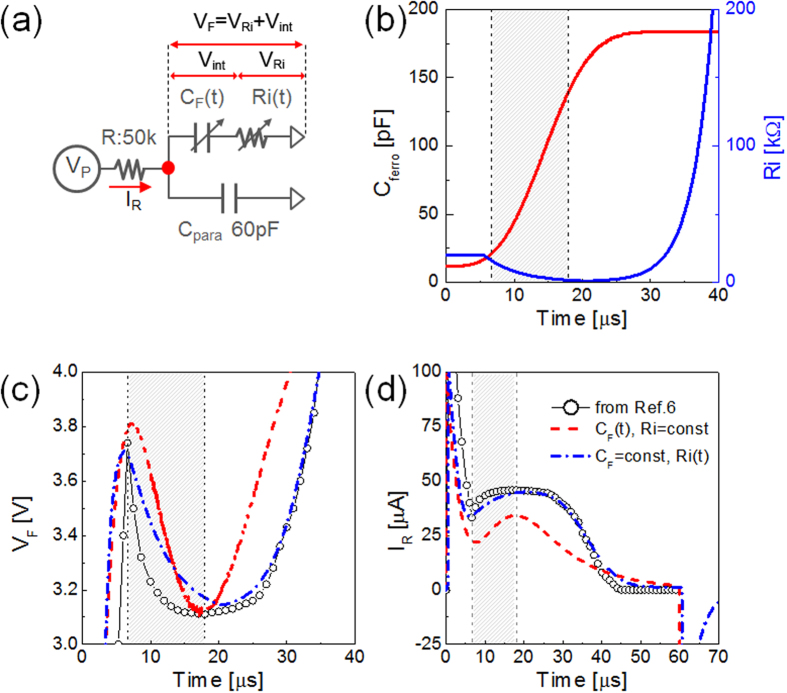
Equivalent circuit simulation results with change in C_F_(t) and R_i_(t) according to the domain nucleation and growth model. (**a**) Schematic circuit diagram for simulation. (**b**) Time-dependent positive capacitance model according to Komogolov-Avrami-Ishibashi theory (red line) and time-dependent R_i_ model (blue line) using in PSPICE simulator. (**c**,**d**) PSPICE simulation result for the variation of the node voltage (V_F_ = V_Ri_ + V_int_) and current flowing through the R (i_R_) during the switching time. Data points (open circle) are reproduced from ref. 6.

Figure 2a describes schematically the equivalent circuit of measurement system for FE switching. Here, the estimated voltage (V_F_) is applied to R_i_ (or ρ according to ref. 6, V_Ri_) and V_int_, meaning that V_F_ = V_Ri_ + V_int_. Therefore, if either voltage, i. e. V_Ri_ or V_int_, decreases with time during the FE switching, the decreased voltage must be added to the external R, and i_R_ must increase accordingly. There could be two probable ways to consider how the V_F_ decreases with time; one is the increase of “positive” capacitance (PC) of the FE layer and the other is the decrease of R_i_ with time. For the former case, conventional definition of C(t) = dQ(t)/dV(t) does not apply because the FE switching charge is not retrieved when the voltage decreases. Nevertheless, for the charging process of a FE switching period, compensating charges flow into the FE layer, so C/A is defined to be 2P_s_/V_c_, where A and P_s_ (= ~75μC/cm^2^, Fig. 2 of on-line Supplementary Information (SI) of ref. 6) are the electrode area and saturation polarization, respectively. Then, the C(t) can be defined from the variation of reversed domain area across the entire electrode according to Komogolov-Avrami-Ishibashi model. With the rapid increase in C(t), V_F_ can decrease under the condition of limited i_R_. In order to prove such claim, the FE film behavior during FE switching is quantitatively simulated by using a PSPICE simulation package, and node voltage (V_F_) and concurrent i_R_ were simulated as a function of time. Red line in Fig. 2b shows the assumed variation of C(t), and red dashed lines in Fig. 2c,d show the simulation results for V_F_ and i_R_, respectively. In this case, capacitor charging current i_C_(t) must be defined as Equation (1), which was then used to calculate the variation of Q with time.


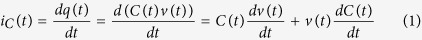


The detailed simulation procedures for calculating V_F_(t), i_R_(t), and Q(t) are described in on-line SI. It was found that variation in voltage across the FE layer (V_int_) is responsible for the variation in V_F_, as described in detail in on-line SI. The V_int_ decreased from a certain value higher than V_c_ to ~V_c_ and rapidly increased again as the switching approached completion, while the V_Ri_ remained constant. This PC model can explain the experimental results quite well. For comparison, experimental data from ref. 6 is also shown (black open dots).

The PSPICE simulation results using varying R_i_(t) model (blue line Fig. 2b) are also appended in Fig. 2c,d using blue dash-dotted lines. Here, R_i_(t) was assumed to vary as R_i_(0)exp(−(t − t_0_)/τ_d_)^β^ according to the soft-dielectric breakdown model across the R_i_^7^, and V_int_ was fixed at V_c_ (=3.1 V from Fig. 2 of on-line SI of ref. 6). R_i_(0), t_0_, τ_d_, and β were assumed to be 20,000 Ω, 5.6 μs, 5 μs, and 1, respectively. When the FE switching was completed, the R_i_(t) was assumed to recover the initial high value with the identical time constant. This is because when the FE switching is completed, the voltage over the FE layer increases eventually to the applied voltage, and no further charge transport across the interface layer (R_i_) is made, which results in the recovery of the original resistance. Even for the heteroepitaxial FE thin film system, there could be extrinsic or intrinsic interfacial (dead) layer at the FE-electrode interface^8^. Although the precise electrical response to the applied voltage of these interfacial layers has been rarely reported, it is reasonable to assume that these layers would show highly non-linear current-voltage characteristics, as presumed in this work, considering the insulating nature of them. The simulation results reproduce the experimental results with surprisingly high accuracy (Fig. 2d), justifying the accuracy of this model. More detailed physical interpretations for R_i_(t) are described in on-line SI.

It would be optimal to directly compare the simulation results using the present method of varying C(t) and R_i_(t) with the simulation results based on the L-K formalism provided by Khan *et al*. (Fig. 4b in ref. 6) to determine which model can more precisely reproduce the experimental results. However, this was unfortunately not feasible because the estimated V_c_ using the L-K formalism was too high (~10 V.) in ref. 6, and, thus, the V_F_ and i_R_ simulation in ref. 6 must have adopted 14 V as the applied voltage, which is very different from the actual experimental situation. Therefore, it can be understood that the observed V_F_↓Q_F_↑ behavior of a single layer FE capacitor during the polarization switching reported in ref. 6 can be explained by these alternative models without involving any conceptual difficulty of NC which indicates that FE layer must pass through the maximum energy state during the polarization switching. More importantly, these alternative models are fundamentally based on the classical nucleation and growth model of reverse domains, which is well-accepted model in ferroelectric community. The simulation also better fits the experimental results than the model based on the L-K theory.

**Figure 3:**
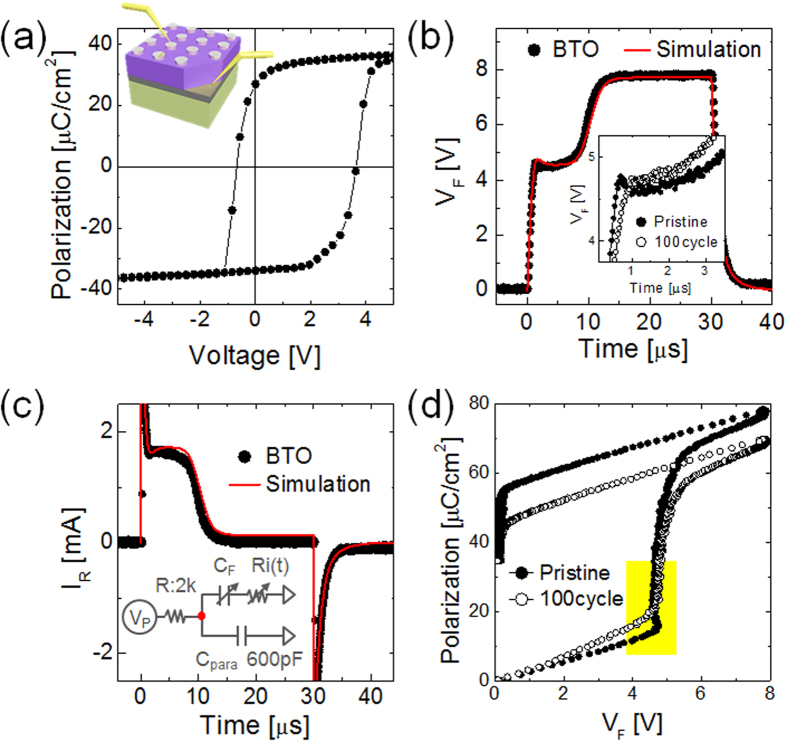
Ferroelectric switching characteristics of epitaxial BTO thin film in DC and AC modes and circuit simulation results with R_i_(t). (**a**) Polarization-voltage hysteresis loops of epitaxial BTO film measured by a commercial ferroelectric tester. (**b**,**c**) PSPICE simulation(red line) and pulsed switching results(closed circle) for the change in the node voltage (V_F_, the detailed view of decreasing V_F_ region shown in inset of (**b**)) and current flowing through the R (i_R_) during the switching time. Inset figure in (**c**) shows the schematic circuit diagram for measurement system. (**d**) Pulsed P-V_F_ curves of BTO film at the pristine state (closed circle) and after 100 switching cycles (open circle) achieved from the integration of i_R_ with time.

In order to further confirm the validity of these alternative models, additional experiments were performed using an epitaxial 150nm-thick BaTiO_3_ (BTO) film (on-line SI for details), which was grown by a pulsed layer deposition technique on SrRuO_3_/DyScO_3_ substrate. Top electrodes were electron-beam evaporated Pt. The P-V loops of the sample is shown in Fig. 3a (inset figure shows the schematic sample structure). Due to the epitaxial strain, the BTO film possessed a 2P_r_ value as high as ~60 μC/cm^2^, +V_c_ of 3.7 V, and −V_c_ of −0.6 V due to the work function mismatch between the top and bottom electrodes and preferential orientation of polarization of the pristine BTO film. To test the charge vs. time and voltage vs. time performances, pulse switching setup was accomplished as shown in the inset of Fig. 3c, where a series resistor of 2 kΩ was connected to the FE thin film sample, and system parasitic capacitance was 600 pF. With this smaller value of R (otherwise circuit noise became too high), the switching time was shortened and a pulse width of 30 μs was long enough to observe the full FE switching. Black closed dots in Fig. 3b,c show the experimental variations in V_F_ and i_R_ as a function of time, according to the format of Fig. 2c,d for easy comparison, and red lines show the fitting results based on the R_i_(t) model mentioned above, where V_c_, R_i_(0), t_0_, τ_d_, and β were assumed to be 3.7 V, 1100 Ω, 0.8 μs, 0.75 μs, and 1, respectively. Here, the experiment was performed on the pristine sample. The model also very well explains this experimental result with a very high accuracy across the entire time span. Inset figure in Fig. 3b shows the detailed view of the decreasing V_F_ region. Black closed symbols in Fig. 3d show the positive portions of the P-V curve of the pristine sample achieved from the integration of i_R_ with time, which is similar to the experimental P-V curves of Khan *et al*. (Fig. 3 of ref. 6.) The region indicated by a yellow box clearly indicates V_F_↓Q_F_↑ behavior, which can be explained by the R_i_(t) model mentioned above. Highly interesting results were found when the identical pulse switching experiments were performed after the BTO capacitor was electrically cycled by 100 times with peak-to-peak voltage of −5V to +5V. In these cases, the portion of V_F_↓Q_F_↑ behavior disappeared (black open circle symbols in inset of Fig. 3b,d) The slight decrease in remnant polarization in Fig. 3d is due to a fatigue effect. These findings strongly suggest that R_i_ disappears during the electrical cycling, perhaps due to the permanent resistance degradation of the interfacial layer. This critical finding indicates that the observed V_F_↓Q_F_↑ behavior of the authors’ BTO film was due to the involvement of R_i_. It is difficult to interpret such disappearance of the V_F_↓Q_F_↑ behavior using a model based on the NC effect, i. e. the slightly fatigued film cannot have any reason why the NC effect is not involved. This is precisely identical to the recent interpretation of the FE switching behavior in experimental results on LiNbO_3_ single crystal film using the varying R_i_(t) model^7^. It may indicate that varying R_i_(t) model, rather than increasing C(t) model, better explains the experimental results. However, Stengel *et al*. reported non-involvement of a dead-layer at a Pt-BTO interface^8^, where the PC model would be suitable.

In summary, the recent experimental results of V_F_↓Q_F_↑ behavior of a circuit containing a large resistor in series with the FE capacitor, which was claimed to be a direct proof of NC behavior of FE layer, were interpreted in alternative methods using a conventional PC model of the FE layer. The critical conceptual standpoint of the PC model is that the FE layer almost always involves a interfacial in-series resistor which played a role as the voltage divider during the fast FE switching experiment, especially under the condition of limited switching charge supply. When this interfacial series resistor is disregarded by repeated cycling in the authors’ own experiments, the experimental findings that might have supported the NC effect disappeared. This could indicate that the experimental results in ref. 6 might also be induced by the involvement of ρ not by the NC effect within the FE layer. The strong point of the alternative models suggested in this work is that reverse domain nucleation and growth during the FE switching are fundamentally assumed, which is the standard understanding for the FE switching well-known to the community. It is also quite notable that Bratkovsky and Levanyuk already indicated that the FE switching mediated by domain wall motion can involve NC effect from the temporary mismatch between the FE switching charge (FE crystal bound charge) and compensating charge induced on the electrode surface^9^,^10^. Therefore, it would be necessary to take a great care to really claim the NC effect from a FE layer given that the domain formation is the preferred path for FE switching from the fundamental energy argument of a FE material.

## Methods

Epitaxial BaTiO_3_ (BTO)/SrRuO_3_ (SRO) bilayer was grown along (001) orientation on (110) DyScO_3_ (DSO) substrate by pulsed laser deposition. The 70nm-thick Pt layer was deposited by e-beam evaporation on top of the BTO layer. And the Pt top electrodes with a cell diameter of 50–100 μm size were patterned by a lift-off process. The characterization of electrical properties, such as current-voltage (I–V) and polarization-voltage (P-V) characteristics, were carried out with using a semiconductor parameter analyzer (HP 4145B) and a ferroelectric tester (TF analyzer 2000), respectively. The time-transient FE switching behavior was analyzed using a pulse generator (HP81110A) and a digital oscilloscope (Tektronix 684C). A square voltage pulse of 8V with a pulse length of 30 μs was programmed to the pulse generator. The FE pulse switching current through the BTO sample was monitored by one of channels of oscilloscope (50Ω), which is connected to the BTO sample in series. At the same time, the voltage across the BTO sample was measured by the other channel (1MΩ), connected to the sample node in parallel.

## Additional Information

**How to cite this article**: Song, S. J. *et al*. Alternative interpretations for decreasing voltage with increasing charge in ferroelectric capacitors. *Sci. Rep.* **6**, 20825; doi: 10.1038/srep20825 (2016).

## Figures and Tables

**Figure 1:**
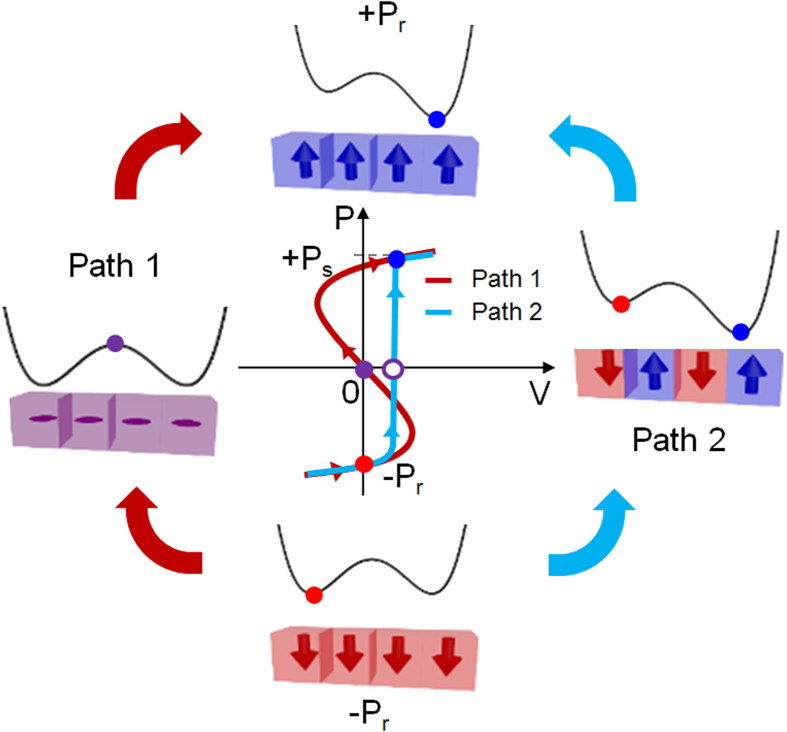
Schematic diagram for variations of polarization state. When a FE film is switched from negative polarization state (−P_r_ state) to positive polarization state (+P_s_ state), it could be occurred via NC state near the V = 0 (path 1, red line) or PC state near the V = V_C_ (path 2, blue line).
